# The impact of individual Cognitive Stimulation Therapy (iCST) on cognition, quality of life, caregiver health, and family relationships in dementia: A randomised controlled trial

**DOI:** 10.1371/journal.pmed.1002269

**Published:** 2017-03-28

**Authors:** Martin Orrell, Lauren Yates, Phuong Leung, Sujin Kang, Zoe Hoare, Chris Whitaker, Alistair Burns, Martin Knapp, Iracema Leroi, Esme Moniz-Cook, Stephen Pearson, Stephen Simpson, Aimee Spector, Steven Roberts, Ian Russell, Hugo de Waal, Robert T. Woods, Vasiliki Orgeta

**Affiliations:** 1 Institute of Mental Health, University of Nottingham, Nottingham, United Kingdom; 2 Division of Psychiatry, University College London (UCL), London, United Kingdom; 3 North Wales Organisation for Randomised Trials in Health (& Social Care), University of Bangor, Bangor, United Kingdom; 4 Whitaker Research Ltd., Bangor, United Kingdom; 5 Institute of Brain, Behaviour and Mental Health, Manchester Academic Health Sciences Centre, Manchester, United Kingdom; 6 Personal Social Services Research Unit, London School of Economics and Political Science, London, United Kingdom; 7 Faculty of Health and Social Care, University of Hull, Hull, United Kingdom; 8 Devon Partnership NHS Trust, Devon, United Kingdom; 9 Dorset Healthcare University NHS Foundation Trust, Dorset, United Kingdom; 10 Research Department of Clinical, Educational and Health Psychology, University College London, London, United Kingdom; 11 Lincolnshire Partnership NHS Foundation Trust, Witham Court, Lincoln, United Kingdom; 12 College of Medicine, Swansea University, Swansea, United Kingdom; 13 Norfolk & Suffolk NHS Foundation Trust, Norwich, United Kingdom; 14 South London and Maudsley NHS Foundation Trust, London, United Kingdom; 15 Dementia Services Development Centre Wales, Bangor University, Bangor, United Kingdom; University of Cambridge, UNITED KINGDOM

## Abstract

**Background:**

Cognitive stimulation therapy (CST) is a well-established group psychosocial intervention for people with dementia. There is evidence that home-based programmes of cognitive stimulation delivered by family caregivers may benefit both the person and the caregiver. However, no previous studies have evaluated caregiver-delivered CST. This study aimed to evaluate the effectiveness of a home-based, caregiver-led individual cognitive stimulation therapy (iCST) program in (i) improving cognition and quality of life (QoL) for the person with dementia and (ii) mental and physical health (well-being) for the caregiver.

**Methods and findings:**

A single-blind, pragmatic randomised controlled trial (RCT) was conducted at eight study sites across the United Kingdom. The intervention and blinded assessment of outcomes were conducted in participants’ homes. Three hundred fifty-six people with mild to moderate dementia and their caregivers were recruited from memory services and community mental health teams (CMHTs).

Participants were randomly assigned to iCST (75, 30-min sessions) or treatment as usual (TAU) control over 25 wk. iCST sessions consisted of themed activities designed to be mentally stimulating and enjoyable. Caregivers delivering iCST received training and support from an unblind researcher.

Primary outcomes were cognition (Alzheimer’s Disease Assessment Scale–cognitive [ADAS-Cog]) and self-reported QoL (Quality of Life Alzheimer’s Disease [QoL-AD]) for the person with dementia and general health status (Short Form-12 health survey [SF-12]) for the caregiver. Secondary outcomes included quality of the caregiving relationship from the perspectives of the person and of the caregiver (Quality of the Carer Patient Relationship Scale) and health-related QoL (European Quality of Life–5 Dimensions [EQ-5D]) for the caregiver.

Intention to treat (ITT) analyses were conducted. At the post-test (26 wk), there were no differences between the iCST and TAU groups in the outcomes of cognition (mean difference [MD] = −0.55, 95% CI −2.00–0.90; *p* = 0.45) and self-reported QoL (MD = −0.02, 95% CI −1.22–0.82; *p* = 0.97) for people with dementia, or caregivers’ general health status (MD = 0.13, 95% CI −1.65–1.91; *p* = 0.89). However, people with dementia receiving iCST rated the relationship with their caregiver more positively (MD = 1.77, 95% CI 0.26–3.28; *p* = 0.02), and iCST improved QoL for caregivers (EQ-5D, MD = 0.06, 95% CI 0.02–0.10; *p* = 0.01). Forty percent (72/180) of dyads allocated to iCST completed at least two sessions per week, with 22% (39/180) completing no sessions at all. Study limitations include low adherence to the intervention.

**Conclusions:**

There was no evidence that iCST has an effect on cognition or QoL for people with dementia. However, participating in iCST appeared to enhance the quality of the caregiving relationship and caregivers’ QoL.

**Trial registration:**

The iCST trial is registered with the ISRCTN registry (identified ISRCTN 65945963, URL: DOI 10.1186/ISRCTN65945963).

## Abstract 2

In a randomized controlled trial, Martin Orrell and colleagues investigate the impact of individual cognitive stimulation therapy (iCST) on cognition, quality of life, caregiver health, and family relationships for people with dementia.

## Author summary

**Why was this study done?:**

- Cognitive stimulation therapy (CST) is a structured group activity programme for people with dementia that has been shown to improve quality of life (QOL) and cognition.
- This therapy is recommended by organisations such as the Alzheimer’s Disease International (ADI) and the UK National Institute for Health and Care Excellence.
- Although CST is becoming more widely available both in the UK and internationally, some people may not have access to groups because groups are not available near their home, they are not able to get to centres offering groups because of transport, health, or mobility problems, or they would prefer not to do group activities.
- This study aimed to look at the potential use and benefits of an adapted version of CST called individual CST (iCST) delivered by a family carer or friend at the person with dementia’s home for 30 min ideally two or three times a week.

**What did the researchers do and find?:**

- The research team produced the iCST programme (including a manual, activity workbook, and materials such as maps and dominoes) in collaboration with people with dementia, carers, health care professionals, and experts.
- In a randomised controlled trial (RCT), 356 pairs of people with dementia and carers were split between two groups; 180 pairs received iCST, and 176 pairs continued with activities, treatments, and services offered as part of usual care but did not receive iCST, to test whether iCST benefits cognition and QOL for people with dementia and mental and physical health for carers.
- The study found that people with dementia receiving iCST did not benefit in terms of cognition or QOL, and neither was there evidence to suggest iCST improved carers’ mental or physical health.
- However, people with dementia in the iCST group reported better relationship quality with their family carer at 26 wk, and carers delivering iCST had better QOL at 26 wk.

**What do these findings mean?:**

- We did not find that iCST improves cognition or QOL for people with dementia.
- Given that iCST appears to have a positive effect on the caregiving relationship and carer well-being, the programme might be a useful part of personally tailored home care packages.

## Introduction

There are an estimated 5.3 million individuals with dementia in the United States, with the number of cases rising each year as the population ages [1]. Family caregivers are an essential source of care with an estimated economic value of $217 billion a year [2].

Dementia caregiving poses unique challenges, and whilst there may be positive aspects, often this role is stressful and can adversely affect the physical and mental health of the caregiver [3,4]. The stress-health model indicates the experience of psychological, behavioural, and physical symptoms associated with dementia are stressful and can reduce quality of life (QoL) for the person and their caregiver [4–6]. In addition, the person’s increasing dependence on others to fulfill basic needs, restructuring of the established relationship, and apathy can all reduce the quality of the relationship between the caregiver and recipient [7–9]. Conflict in the caregiving relationship is a risk factor for deterioration of functioning in the person with dementia and presentation to services [10], and there is evidence to suggest that maintenance of this relationship may facilitate a good QOL, slow the progression of cognitive and functional decline, and delay institutionalisation [11,12].

There is growing recognition that psychological interventions can improve QOL and should be more widely available. Amongst those that enhance the QOL of people with dementia, cognitive stimulation therapy (CST) has a robust evidence base [13–15] and has been shown to improve patient QoL and cognition and also to be cost-effective [16]. An extended programme of maintenance CST (CST plus an additional 24 weekly sessions) was found to improve QoL [17]. Cognitive stimulation is based on the theory of “use it or lose it,” whereby mental stimulation may counter or slow cognitive decline, and evidence that activation of neurons may enhance neuronal function and survival [18,19]. CST sessions are designed to provide general stimulation of a range of cognitive skills through enjoyable activities in a social setting, although language appears to be particularly affected [20]. Further investigation of CST’s impact on QoL indicates that the domains of energy level, memory, ability to do chores, and relationship with caregiver are most responsive to improvement, and that improvements in QoL may be mediated by improvements in cognition [21]. Typically, CST is delivered in day centres or residential care facilities, without the family caregiver. The stress-health model suggests that improvements in QOL and cognition from CST may improve caregiver outcomes, but few studies have examined this [5].

Many of the therapies currently available are directed at either caregivers or people with dementia, but a meta-analysis of psychological interventions for caregivers suggests that interventions are less efficacious when they target caregivers alone [22]. Home-based, multi-component dyadic interventions, engaging both the caregiver and the person with dementia, have been found to yield a range of benefits, including reduction in behavioural symptoms [23], reduction in negative caregiver reactions [24], and reduction in nursing home admissions [25]. The current evidence on both caregiver-focused and dyadic interventions also suggests that delivery one to one is more effective than in a group [6,22].

This suggests that a home-based, one-to-one version of CST led by a family caregiver may yield benefits for both the person and the caregiver. Few studies have focused on the use of cognitive stimulation–based programmes delivered in the home, and CST has never been directly adapted for use in this context. However, a small study of home-based memory management by family caregivers with psychoeducation improved memory in the person with dementia, improved caregiver well-being, and reduced care home admissions by 18 mo follow-up [26]. Similar benefits in cognition in people with dementia and caregiver well-being have been reported in other studies [27,28]. A further potential benefit of developing a home-based version of CST would be increasing the accessibility of the intervention for people unable to get to groups due to health/mobility problems, lack of groups in the local area, or preference not to participate in group activities [29].

The aim of the individual CST (iCST) trial was to investigate the primary outcomes of whether family caregiver-delivered CST improves (i) cognition and QoL of people with dementia and (ii) mental and physical health of caregivers. We hypothesised iCST may elicit cognitive benefits for the following reasons: (i) the programme provides mental stimulation through multisensory activities exercising a range of cognitive skills (e.g., memory, communication) in an environment that supports learning [30]; (ii) like CST, iCST focuses on implicit memory, which tends to be maintained longer than explicit memory and, moreover, responds to stimulation [31]; (iii) discussion of new thoughts and ideas, and making associations (key principle of iCST) stimulates language [30]. The existing evidence on group CST and other individual family-led cognitive interventions [13,26–28] also supports the choice of cognitive change as a primary outcome in this trial. QoL was also chosen as a primary outcome because (i) improvements in cognitive function appear to mediate improvements in QoL; therefore, if iCST benefitted people cognitively as predicted, we expected to observe an associated positive impact on QoL, and (ii) cognitive stimulation has consistently been found to improve QoL [15].

Secondary outcomes for people with dementia included behavioural and psychological symptoms, activities of daily living, depressive symptoms, and the quality of the caregiving relationship. We posited that iCST may improve the caregiving relationship because (i) improving cognition may help people communicate more effectively with their caregiver, which is associated with higher relationship satisfaction [11], and (ii) the programme provides an opportunity for people and their caregivers to participate in enjoyable activities together [32,33]. In addition, enhancing the quality of the caregiving relationship may also improve QoL for the person with dementia [11], supporting selection of QoL as a primary outcome in this trial.

For caregivers, health-related QoL, mood symptoms, resilience, and relationship quality were secondary outcomes. We hypothesised that participating in activities together may help caregivers develop or maintain a closer relationship with the person they are caring for, and focusing these kinds of positive aspects of caregiving may improve well-being and reduce stress and burden [34]. Experience of enjoyment through caregiving could serve as a coping resource; therefore, we anticipated participating in iCST may positively impact resilience [34].

## Methods

### Ethics statement

Ethical approval was obtained through the East London 3 Research Ethics Committee (ref no. 10/H0701/71). The study was registered as a clinical trial (ISRCTN 65945963). Participants gave informed consent in accordance with the UK Mental Capacity Act (2005) [35].

### Trial design and setting

**Fig 1:**
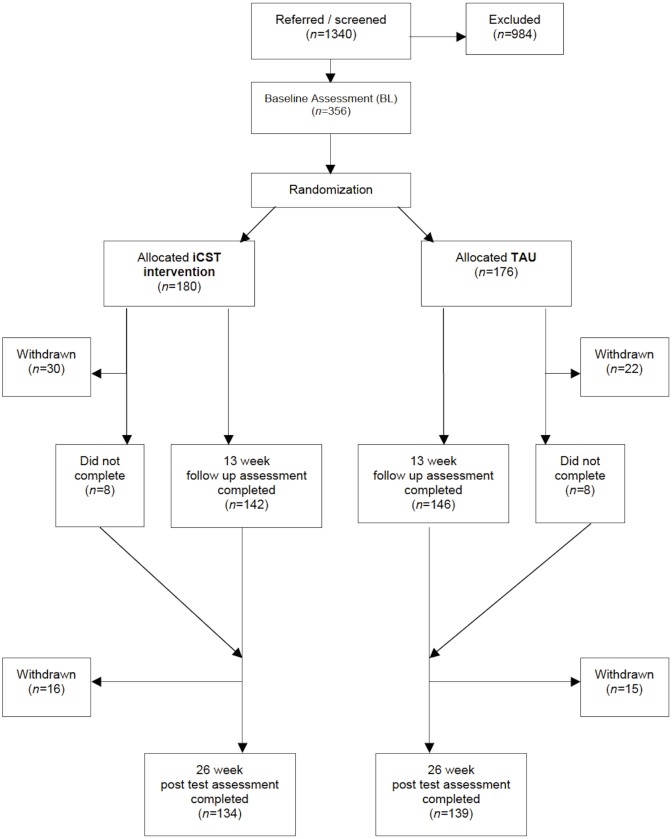
Participant flow through the trial. “Withdrawn” indicates participants’ withdrawal from trial and all associated research activities. “Did not complete” indicates participants who missed 13-wk follow up assessment but returned for the 26-wk post test.

We conducted a single-blind, two-group pragmatic randomised trial of iCST over 25 wk against treatment as usual (TAU) over 25 wk (Fig 1). The full protocol is described elsewhere [29]. The trial operated from eight centres across the UK (London, Bangor, Dorset, Devon, Hull, Lincolnshire, Manchester, and Norfolk & Suffolk). From April 2012 to July 2013, recruitment took place in a variety of community settings including National Health Service (NHS) memory clinics, community mental health teams (CMHTs) for older people, and associated outpatient clinics.

### Recruitment

Participants were recruited in the community from memory clinics, CMHTs, outpatient clinics, day centres, and voluntary organisations such as the Alzheimer’s Society. The aim of the project was briefly described to potential participants by members of the research and clinical team, and permission for them to be contacted by local researchers was obtained prior to further contact. Research assistants discussed the project and provided full details to participants, answered any questions related to the project, and, if participants agreed, undertook written informed consent.

### Participants

Participants met the criteria for dementia of the Diagnostic and Statistical Manual of Mental Disorders [36], had dementia of mild to moderate severity (Mini Mental State Examination [MMSE] score ≥10) [37], had some ability to communicate and understand, and were able to see and hear well enough to participate in activities. In addition, each participant lived in the community, had no major illness affecting their participation, and had a caregiver (relative or friend) able to deliver the intervention and act as an informant for the assessments. If caregivers were not able to support the person in the delivery of iCST sessions, they were not recruited into the trial.

### Sample size

Based on previous studies [13] and the Cochrane Review of cognitive stimulation [15], we conservatively powered the trial to detect a mean difference (MD) between iCST and TAU of 0.35 on the Alzheimer’s Disease Assessment Scale–Cognitive Subscale (ADAS-Cog) [38]. To yield 80% power when using a *t* test with a two-sided 5% significance level, and assuming 15% attrition, we needed a sample size of 306 dyads. As actual attrition was 23% (83/356), we more than compensated by increasing the target to 356 dyads during the trial. One hundred eighty dyads were allocated to iCST, and 176 received TAU.

### Randomisation and blinding

After baseline (BL) assessment, we allocated dyads (people with dementia and caregivers) at random between iCST and TAU groups in the ratio 1:1. To prevent subversion, we used dynamic allocation [39] and the web-based randomisation service managed by North Wales Organisation for Randomised Trials in Health, an accredited UK Clinical Trials Unit. We stratified participants by centre and use of cholinesterase inhibitors. We concealed allocations from researchers conducting 13-wk mid-point and post-test (26 wk) assessments. To assess the success of blinding, these researchers recorded the perceived allocation of each participant at each assessment.

### Intervention

The iCST program was delivered at home by a caregiver in regular contact with the person with dementia (see Text A in S1 Appendix). The iCST package included a manual containing guidance on sessions, key principles of iCST, and ideas for activities; an activity workbook with paper resources for activities; and a toolkit of additional items such as playing cards, dominoes, sound activity compact discs (CDs), and maps. Dyads engaged in up to three, 30-min sessions of structured cognitive stimulation through themed activities (e.g., word games, current affairs, being creative, see Table A in S1 Appendix for all iCST themes) per week over 25 wk (maximum of 75 sessions). A sample session is shown in Box 1 with details of the procedure and content of the first session of the iCST program: “My Life (Life History) Part I.” All sessions follow the same general structure, and a selection of activities is offered for each theme with two levels of difficulty; Level A activities were intended to be less cognitively demanding and more discussion based, whilst Level B activities were more cognitively challenging (see Box 1 for examples).

**Box 1. Sample iCST session: My life (life history) part I**

**Introduction/warm-up (5–10 min)**
- Discuss orientation information such as the day, date, and weather using aids such as the newspaper and surroundings (e.g., looking out of the window to prompt comments about the weather). The purpose of this introduction is to orientate the person to the here and now.
- Talk about something currently happening; this could be national or local news, events in the community, or personal events such as birthdays and special occasions (e.g., birth of a new child in the family). Discussion can be prompted using newspapers, online news articles, and documents such as invitations, etc.

**Main activity (20–25 min)**
- **Level A:** Look at old and recent family photographs of family and friends. Prompt with questions about shared traits of family members, interesting stories about them, tips to maintain good relationships, or advice you would give about having a family.
- **Level B:** Record family history in a family tree. Add details for each generation such as relationships, birthdays, and place of birth. Share stories about the family and talk about how people’s lives have changed over the years.

**Materials from iCST activity workbook:** An example of a family tree and a template to give dyads a starting point for this activity, which can be used for the Level B option. No materials are provided for Level A, as this activity requires dyads to provide photographs from their own personal collection.

**Materials from the iCST toolkit:** The colour pencils could be used to design the family tree, the UK and world maps could be used to prompt discussion about places of birth and location of family members, and the magnifying card could be used to see finer details on photographs or written records such as birth certificates, which may be used as cues in the activity.

The development of the programme was rigorous, adhering to the UK Medical Research Council framework [40]. The program was based on a modified CST manual, the recent Cochrane review of cognitive stimulation [15], an individual manualised programme of reality orientation [28], and consultation with caregivers, people with dementia, and professionals in dementia care in a series of focus groups, consensus work, and field testing [41].

### Treatment adherence, caregiver training, and support

We followed previous studies [42] applying the treatment integrity model developed and expanded on by Lichstein, Riedel, and Grieve [43]. Intervention pairs were visited at home by a dementia researcher as soon as possible after randomisation to provide them with the iCST materials and train them in the iCST approach. Dementia researchers included mental health nurses, clinical psychologists, occupational therapists, and research assistants. All researchers who provided support to family caregivers received standardised training and followed a treatment protocol. The standardised training package researchers delivered to caregivers taught them how to use the iCST manual and activity workbook and implement the key principles of iCST. Clips from the group CST training DVD, “Making a Difference 2” [44], were shown to demonstrate good practice. After learning about the iCST approach, the caregiver delivered the first session with support from the researcher, who provided assistance and feedback. Typically training visits lasted between 60 and 90 min. Caregivers also received up to 10 h of support from the dementia researcher during the trial, including telephone support (initially weekly) and two scheduled monitoring home visits (monitoring visit 1 [MV1] and monitoring visit 2 [MV2]). If the key family caregiver was unable to continue delivering iCST, an appropriate caregiver was substituted if possible.

### TAU

As the trial examined the effects of adding iCST to TAU, the regular services offered were the same in both groups. Not surprisingly, TAU varied between and within centres and over time (see Text B in S1 Appendix for more information). Standard best practice methods around pragmatic RCTs were followed, and it was expected that both the iCST and TAU groups had access to a similar range and similar types of mentally stimulating activities outside the research trial, for instance non CST-based group activities offered by day centres, hobbies, gardening, support groups, or visits to places of interest. In terms of use of CST, participants who had attended CST groups in the 3 mo before recruitment into the trial were considered ineligible. Sites were asked to record any instances of engagement in CST or other activities offered by local services during the trial. As far as we were aware, participants were unlikely to have access to any comparable individual cognitive stimulation interventions, as this type of structured therapy is generally not available in the UK. General service use and medication were recorded in both groups.

### Assessment and measures

We completed primary and secondary measures at BL, 13 wk after BL (mid-point), and 26 wk after BL (post-test and primary endpoint).

### Outcome measures for the person with dementia

The ADAS-Cog was selected to measure the primary outcome of cognition and consists of 11 tasks assessing memory, language, praxis, attention, and other cognitive abilities [38]. The scale is widely used and psychometrically sound, with good reliability and validity. The measure we selected to assess QoL was the Quality of Life Alzheimer Disease Scale (QoL-AD), which has good validity and reliability [45]. Secondary outcomes included dementia-specific QoL (Dementia Quality of Life [DEMQOL]) [46], neuropsychiatric symptoms (Neuropsychiatric Inventory [NPI]) [47], functional ability (Bristol Activities of Daily Living Scale [BADLS]) [48], and depressive symptoms (Geriatric Depression Scale [GDS-15]) [49].

Quality of the carer–patient relationship (Quality of the Carer–Patient Relationship Scale [QCPR]) was an additional measure included in response to data from the field-testing phase of the trial, which indicated that the caregiving relationship may benefit as a result of participating in the intervention [50,51]. The QCPR is split into two subscales: criticism and warmth. As a covariate, we graded severity of dementia using the Clinical Dementia Rating Scale (CDR) [52].

### Outcome measures for the caregiver

The primary outcome for caregivers was mental and physical health (well-being) measured by the Short Form-12 Health Survey (SF-12) [53]. Secondary outcomes were anxiety and depressive symptoms (Hospital Anxiety and Depression Scale [HADS]) [54], health-related QOL (European Quality of Life–5 Dimensions [EQ-5D]) [55] to which we applied societal weights [56], resilience (Resilience Scale [RS-14]) [57], and quality of the caregiving relationship (QCPR) [50].

### Caregiver adherence measures

Caregivers in the iCST group completed self-report questionnaires at the set-up visit, MV1, and MV2, which required them to rate their confidence (4-point scale: very little, fair, good, very confident) in delivering iCST, quality of support (5-point scale A: excellent, very good, good, fair, poor), knowledge of iCST (see 5-point scale A), successful engagement (5-point scale B: all of the time, most of the time, some of the time, a little of the time, none of the time), and application of specific techniques (opinions rather than facts, developing ideas in a sensitive manner, incorporating person’s interests into programme, adapting sessions for the person) and skills in delivering the sessions (see 5-point scale B). The questionnaire was developed specifically for use in this trial to measure treatment integrity and whether the intervention was carried out as intended [58].

### Anticipated risks

As there are no documented harmful side effects from participating in CST, we expected few adverse events in this trial [13]. Sites recorded and reported serious adverse events (SAEs) to the Chief Investigator.

### Statistical analysis

We analysed all available data by treatment allocated, following the principles of Intention to Treat (ITT). Statisticians performing the main analysis were blind to randomised intervention assignment. We used analysis of covariance (ANCOVA) to estimate the differences between iCST and TAU groups for people with dementia in primary and secondary outcomes at the 13-wk mid-point and post-test (26 wk). The model adjusted for covariates expected to influence outcome variables, including BL score on the outcome measures, the age of the person with dementia, and relationship with the caregiver. The fixed factors were gender, marital status, and use of anti-cholinesterase inhibitors, and centre was a random factor. We used a similar ANCOVA for primary and secondary outcomes for caregivers at the 13-wk mid-point and post-test with covariates of BL scores, age of caregiver, and relationship with the person with dementia, fixed factors of gender and marital status, and random factor of centre. Effect sizes were calculated using Cohen’s d.

### Adherence analyses

Carer adherence data were collected and paired *t* test analysis performed to compare the differences between the set-up visit, MV1, and MV2.

### Exploratory analyses

To analyse adherence, linear regression was used to assess the relationship between the follow-up outcome measures and the number of iCST sessions attended after adjusting for BL outcome measures. This method was considered more efficient for an exploratory analysis than either defining an average number of sessions to complete a week or predefining a number of sessions to be “enough” of the therapy.

Any participants who did not provide any data post-test or at the 13-wk mid-point were not included in the analysis. If a participant had less than 20% of the items missing for a scale, then we prorated the scores for that measure [59]. This left fewer than ten total scores missing, and for these we then used multiple imputation based on a linear regression method. The number of imputations created was based on the percentage of missing data.

## Results

### Preliminary analyses

There were no differences between the two groups at BL on clinical and demographic factors (Table 1). Three hundred and fifty-six pairs participated in the trial. Recruitment was complete by July 2013, with the final post-test assessments complete by February 2014. Analysis by treatment allocated included 134 iCST and 139 TAU dyads. Twenty-three percent (83/356) of the total sample (75/356, 21% excluding deaths) dropped out by post-test. Rates of attrition in the iCST (46/180, 26%) and TAU (37/176, 21%) groups were not significantly different. Average BL MMSE scores were similar (iCST = 21.12, standard deviation [SD] = 4.48; TAU = 21.33, SD = 4.11). Characteristics of completers and noncompleters are provided in Table B in S1 Appendix, which shows no differences at BL. Reasons for dropouts are shown in Table C in S1 Appendix. Out of the 46 withdrawals in the iCST group, 18 did not wish to continue (sometimes noting they were too busy), and 28 were unable to participate largely because of ill health or having relocated. Six from the TAU group withdrew because they were not allocated to iCST, amounting to only one in 30 TAU participants. Seventy percent of the sample had mild dementia (CDR score = 1). In relation to other activities, there was no difference between the intervention and TAU groups at BL or post-test in terms of day centre attendance, lunch club attendance, or education classes.

**Table 1:** Baseline characteristics of person with dementia and caregiver.

Characteristic Person with dementia	Total (%) (*n* = 356)	iCST (%) (*n* = 180)	TAU (%) (*n* = 176)
Female	165/356 (46)	83/180 (46)	82/176 (47)
Ethnicity: white	331/356 (93)	164/180 (91)	167/176 (95)
Marital status: married/cohabiting/civil partnership	252/356 (71)	125/180 (69)	127/176 (72)
Lives with spouse/partner	225/356 (63)	113/180 (63)	112/176 (64)
Highest level of education school leaver (14–16 y)	213/356 (60)	113/180 (63)	100/176 (57)
Anti-cholinesterase inhibitors	270/356 (76)	136/180 (76)	134/176 (76)
**Caregiver**			
Female	261/356 (73)	135/180 (75)	126/176 (72)
Ethnicity: white	329/356 (92)	164/180 (91)	166/176 (94)
Marital status: married/cohabiting/civil partnership	297/356 (83)	149/180 (83)	148/176 (84)
Highest level of education school leaver (14–16 y)	156/356 (44)	79/180 (44)	80/176 (45)

iCST, individual cognitive stimulation therapy; TAU, treatment as usual.

### Researcher ratings of perceived group allocation

The response rate for the researcher perceived group allocation questionnaires (see Table D and Table E in S1 Appendix) was high at the 13-wk mid-point (92%, 264/288) and post-test (93%, 255/273). At both assessment time points, most blinded researchers were not able to identify whether dyads were receiving iCST or TAU (60%, 160/264 at mid-point and 57%, 145/255 post-test). Overall, again at both time points, only 23% were able to accurately predict which group the dyads had been allocated to, with the remainder judging incorrectly.

### Outcomes for person with dementia

The primary outcomes of cognition and QOL (ADAS-Cog, QoL-AD) were not statistically significant at the 5% level between iCST group and TAU group at the 13-wk mid-point or the primary end point post-test (Table 2). However, there was a significant improvement in QCPR total score for the iCST group relative to the TAU group, with an MD of 1.77 (95% CI 0.26–3.28; *p* = 0.02) and effect size of 0.32. No significant differences between groups were detected for activities of daily living, depression, or behavioural and psychological symptoms. There were no differences in primary or secondary outcomes at the 13-wk mid-point (Table 3). Summaries of outcomes and change from BL scores are provided in Tables F and G in S1 Appendix, respectively.

**Table 2:** Outcome measures at 26-wk post-test by iCST versus TAU: Complete case analysis, adjusting for BL outcome measures, marital status, centre, age, and anticholinesterase inhibitors.

26-wk post-test person with dementia	iCST (*n* = 134)	TAU (*n* = 139)	MD	95% CI of MD	*p* value
ADAS-Cog	20.03	20.58	−0.55	(−2.00–0.90)	0.45
QoL-AD	37.90	37.92	−0.02	(−1.04–1.00)	0.97
DEMQoL	94.45	94.14	0.31	(−1.62–2.22)	0.79
NPI [P]	8.10	8.42	−0.32	(−2.78–2.12)	0.79
GDS-15	3.29	3.31	−0.02	(−0.51–0.47)	0.94
QCPR Total*	57.42	55.65	1.77	(0.26–3.28)	0.02*
MMSE	19.63	20.10	−0.47	(−1.26–0.30)	0.23
BADLS [P]	11.91	12.57	−0.66	(−2.07–0.75)	0.36
QoL-AD [P]	32.45	32.00	0.45	(−0.71–1.60)	0.45
DEMQoL [P]	99.67	97.94	1.73	(−0.61–4.07)	0.15
**Caregiver**					
SF-12 Physical component	49.57	49.11	0.46	(−1.21–2.13)	0.59
SF-12 Mental component	48.44	48.31	0.13	(−1.65–1.91)	0.89
HADS Anxiety	6.09	6.30	−0.21	(−0.94–0.52)	0.57
HADS Depression	4.16	4.67	−0.51	(−1.09–0.08)	0.09
EQ-5D health state today	78.20	76.99	1.21	(−2.14–4.57)	0.48
EQ-5D calculated utility value*	0.82	0.76	0.06	(0.01–0.10)	0.01*
RS-14	83.42	81.85	1.58	(−0.37–3.52)	0.11
NPI Carer distress	3.13	3.22	−0.09	(-0.55–0.37)	0.70
QCPR total	59.65	60.21	−0.56	(−1.93–0.82)	0.43

Note: Complete case data are presented owing to little difference between this and imputed data results.

* Significant difference.

ADAS-Cog, Alzheimer’s Disease Assessment Scale–Cognitive; BADLS, Bristol Activities of Daily Living Scale; DEMQoL, Dementia Quality of Life, EQ-5D, European Quality of Life–5 Dimensions; GDS, Geriatric Depression Scale; HADS, Hospital Anxiety and Depression Scale; iCST, individual cognitive stimulation therapy; MD, mean difference; MMSE, Mini Mental State Examination; NPI, Neuropsychiatric Inventory; [P], Proxy rated measure; QCPR, Quality of the Carer–Patient Relationship; QoL-AD, Quality of Life Alzheimer Disease; RS-14, Resilience Scale; SF-12, Short Form-12 Health Survey; TAU, treatment as usual.

**Table 3:** The means (and 95% CIs) comparing the iCST and TAU for person with dementia outcome measures at 13-wk mid-point after adjusting for marital status, centre, age, and anticholinesterase inhibitors. (Complete case data are presented due to little difference between this and imputed data results).

13-wk mid-point person with dementia	Missing	iCST (*n* = 142)	TAU (*n* = 146)	MD	95% CI of MD	*p* value
ADAS-Cog	10	22.00	21.71	0.29	(−1.10–1.68)	0.68
QoL-AD	4	38.40	38.54	−0.14	(−1.12–0.84)	0.78
DEMQoL	11	91.72	92.05	−0.33	(−2.31–1.65)	0.74
NPI [P]	2	12.27	13.72	−1.45	(−3.68–0.76)	0.20
GDS-15	12	3.27	3.36	−0.09	(−0.56–0.38)	0.71
QCPR total	7	56.62	55.52	1.10	(−0.15–2.35)	0.09
MMSE	3	20.32	20.16	0.16	(−0.60–0.92)	0.69
BADLS [P]	1	12.73	12.93	−0.20	(−1.44–1.04)	0.75
QoL-AD [P]	3	32.66	31.91	0.75	(−0.27–1.77)	0.15
DEMQoL [P]	3	99.28	98.73	0.55	(−1.70–2.80)	0.64
**Caregiver**						
SF-12 Physical component	0	50.51	50.57	−0.06	(−1.45–1.33)	0.93
SF-12 Mental component	0	47.59	48.30	−0.71	(−2.34–0.92)	0.39
HADS Anxiety	1	10.47	10.31	0.16	(−0.81–1.15)	0.74
HADS Depression	1	6.34	6.05	0.29	(−0.35–0.91)	0.37
EQ-5D health state today	1	4.13	4.27	−0.14	(−0.67–0.39)	0.60
EQ-5D calculated utility value*	1	77.55	77.00	0.55	(−2.59–3.69)	0.73
RS-14	1	0.81	0.79	0.02	(−0.02–0.06)	0.19
NPI Carer distress	0	83.35	83.41	−0.06	(−1.63–1.51)	0.94
QCPR total	2	3.16	3.15	0.01	(−0.43–0.43)	0.99

* Significant difference at 5% level.

ADAS-Cog, Alzheimer’s Disease Assessment Scale–Cognitive; BADLS, Bristol Activities of Daily Living Scale; DEMQoL, Dementia Quality of Life, EQ-5D, European Quality of Life–5 Dimensions; GDS, Geriatric Depression Scale; HADS, Hospital Anxiety and Depression Scale; iCST, individual cognitive stimulation therapy; MD, mean difference; MMSE, Mini Mental State Examination; NPI, Neuropsychiatric Inventory; [P], Proxy rated measure; QCPR, Quality of the Carer–Patient Relationship; QoL-AD, Quality of Life Alzheimer Disease; RS-14, Resilience Scale; SF-12, Short Form-12 Health Survey; TAU, treatment as usual.

### Caregiver outcomes

There were no differences in the primary outcome of functional health status (well-being) on the SF-12 (Table 2). The EQ-5D calculated utility value for the caregiver was significantly better post-test for the iCST group, with an MD of 0.06 (95% CI 0.01–0.10, *p* = 0.014) and effect size of 0.25. Reduced HADS depression score in the iCST group at post-test (−0.51, 95% CI −1.09–0.08, *p* = 0.09) did not reach significance. No differences in any other outcomes were found at the 13-wk mid-point (Table 3).

### Adherence analysis

One hundred and seventy-three carers completed questionnaires at setup, 141 at MV1, and 124 at MV2. Some carers did not complete the questionnaires, as they dropped out before the monitoring visit. At the set-up visit, carers scored their knowledge of iCST at 3.14 (out of 4), and it had improved to 3.58 at MV2 (MD = 0.371, 95% CI 0.285–0.457, *p* ≤ 0.001). In addition, carers’ confidence in delivering iCST improved from 2.98 (out of 4) at MV1 to 3.23 at MV2 (MD = 0.25, 95% CI 0.173–0.327, *p* ≤ 0.001).

Overall, carers stated they felt that they had very good abilities to apply iCST key principles and skills related to the intervention, with scores ranging from 3.76 to 3.96 at MV1 and improving between MV1 and MV2. These included “focusing on opinions rather than facts” (MD = 0.89, 95% CI 0.038–0.139, *p* ≤ 0.001), “developing ideas in a sensitive manner” (MD = 0.145, 95% CI 0.082–0.208, *p* ≤ 0.001), “incorporating their relative’s personal interests in the activities” (MD = 0.153, 95% CI 0.078–0.229, *p* ≤ 0.001), and “adapting the sessions to accommodate their relative’s abilities (MD = 0.089, 95% CI 0.017–0.160, *p* = 0.016). At setup, 71% (122/173) carers anticipated they would need little or no support in delivering the intervention. Set-up training, telephone support, and monitoring visits were well received by most carers, with 81% (114/141) of ratings being “good” or “excellent.”

The majority of carers (83%, 144/173) felt they would be able to engage in iCST with the person with dementia most or all of the time. However, from the carer’s perspective, there appeared to be no significant differences in the person with dementia’s level of engagement from MV1 to MV2 (MD = −0.016, 95% CI −0.067–0.034, *p* = 0.529).

In terms of number of sessions completed, only 40% (72/180) of dyads allocated to iCST completed at least two sessions per week, in line with the expected minimum for effectiveness, with 22% (39/180) completing no sessions at all.

### Exploratory analyses

#### Outcomes for people with dementia

Twenty-two percent of the sample (39/180) in the iCST group did not complete any of the sessions, but 51% (91/180) were able to complete more than 30 sessions over 25 wk. Forty percent completed two to three sessions per week. When the linear regression model was fitted, there was no relationship between the number of sessions attended and the primary outcomes at any time point. However, the total number of sessions was associated with a significant improvement in the quality of the caregiving relationship from the person with dementia’s viewpoint (QCPR total, *p* = 0.003; QCPR criticism, *p* = 0.001). This result was consistent for QCPR total after regression analysis with imputed data. The imputation was not conducted for QCPR criticism at post-test, as no data were missing (Table 4). At the 13-wk mid-point, QCPR lower criticism had a significant association with higher number of sessions received (*p* = 0.004; results shown in Table H in S1 Appendix).

**Table 4:** Regression of outcome measures at 26-wk post-test on the number of sessions of iCST attended, adjusting for BL outcome measures, marital status, centre, age, and anticholinesterase inhibitors.

Observed data, 26 wk post-test							
Person with dementia	coefficient	95% CI	*p* value	Caregiver	coefficient	95% CI	*p* value
ADAS-Cog	−.013	(−0.040, 0.015)	.361	SF-12 Physical component	.018	(−0.013, 0.049)	.275
QoL-AD	.008	(−0.011, 0.027)	.402	SF-12 Mental component	.017	(−0.016, 0.050)	.338
DEMQoL	.007	(−0.029, 0.044)	.691	HADS total	−.020	(−0.042, 0.001)	.064
NPI total	−.002	(−0.048, 0.044)	.927	HADS Anxiety	−.007	(−0.021, 0.006)	.283
GDS-15	.001	(−0.008, 0.011)	.815	HADS Depression*	−.013	(−0.025, −0.003)	.018*
QCPR total*	.043	(0.015, 0.071)	.003*	EQ-5D health state today	.020	(−0.043, 0.083)	.525
MMSE	.006	(−0.009, 0.021)	.455	EQ-5D calculated utility value	.0007	(−0.000, 0.002	.090
BADLS [P]	−.015	(−0.041, 0.011)	.264	RS-14	.023	(−0.013, 0.061)	.232
QoL-AD [P]	.012	(−0.010, 0.034)	.269	NPI Carer distress	−.005	(−0.014, 0.003)	.228
DEMQoL [P]	.013	(−0.031, 0.058)	.558	QCPR	−.006	(−0.032, 0.020)	.673

* Significant difference.

ADAS-Cog, Alzheimer’s Disease Assessment Scale–Cognitive; BADLS, Bristol Activities of Daily Living Scale; DEMQoL, Dementia Quality of Life, EQ-5D, European Quality of Life–5 Dimensions; GDS, Geriatric Depression Scale; HADS, Hospital Anxiety and Depression Scale; iCST, individual cognitive stimulation therapy; MMSE, Mini Mental State Examination; NPI, Neuropsychiatric Inventory; [P], Proxy rated measure; QCPR, Quality of the Carer–Patient Relationship; QoL-AD, Quality of Life Alzheimer Disease; RS-14, Resilience Scale; SF-12, Short Form-12 Health Survey.

#### Outcomes for caregivers

HADS depression scores showed a significant reduction in the iCST group post-test (*p* = 0.018) for caregivers who had participated in a higher number of sessions (Table 4). This was supported by the imputation analysis (*p* = 0.013).

### SAEs

Twenty-five SAEs occurred in the iCST group and 26 in the TAU group, of which 44 related to people with dementia and seven involved caregivers. The most frequent reported category of SAE was “hospitalisation” (63%, 32/51), of which there were 16 instances in each group. There were more deaths in the TAU group (8/10) than the iCST group (2/10). Five SAEs categorised as “life-threatening” were recorded in total, three of which occurred in the iCST group, and four “medically significant” SAEs occurred in the iCST group. For three people with dementia, two SAEs were reported, which were hospitalisations followed by death. We judged that none were definitely, probably, or possibly related to treatment received within the trial, either iCST or TAU.

## Discussion

We undertook a pragmatic RCT to evaluate the impact of a programme of individual, home-based CST on cognition and QoL of people with dementia and mental and physical health of caregivers. No significant differences were found between the iCST and TAU groups for the primary outcomes of cognition and QoL for people with dementia and mental and physical health for caregivers. iCST appeared to enhance the quality of the caregiving relationship from the person with dementia’s perspective. In addition, the caregivers in the intervention group benefitted in terms of improvements in QoL (EQ-5D). Analyses incorporating level of adherence to the iCST programme (number of sessions completed) revealed that people with dementia who participated in more sessions were more likely to experience gains in the relationship with their caregiver at the primary end point of the study (26 wk), and caregivers who completed more sessions had fewer depressive symptoms. The EQ-5D demonstrated improvements in QoL for caregivers, but there was no difference in general health status (SF-12). The discrepancy in findings between these two measures may be related to inherent differences in the measures or their sensitivity to change.

With a total of 356 participating pairs, to our knowledge, this is the largest trial of a CST-based approach. The trial also represents an innovation in CST, as previously the intervention had been delivered only in groups without caregivers. A potential limitation of this trial is the low levels of adherence to the intervention. Because less than half of the iCST group completed at least two sessions per week (72/180, 40%) and 22% (39/180) did not complete any sessions, the power of the study to identify significant differences in outcomes between the iCST and TAU groups may have been compromised.

Data from the development phase of the trial and qualitative data gathered from post-trial interviews may yield insight into the reasons for low levels of adherence. The principal reason for nonadherence in the field-testing study was difficulty fitting iCST into a busy schedule [51]. In post-trial interviews challenges that hindered adherence included difficulty engaging the person in the activities, which in some cases was due to the activities being too easy, poor health of the person with dementia, and having a negative outlook about the progressive nature of dementia, which may have affected caregiver motivation to deliver the intervention [60]. It may be that the intervention is simply not suitable for all and that there are particular characteristics of (i) the context in which the intervention is delivered (e.g., relationships, health, life events), (ii) the person delivering the intervention (e.g., motivation, personality), and (iii) the person receiving the intervention, which act as mediators of successful engagement in and adherence to the programme. More detailed investigation of these factors in order to discern their impact on adherence would be useful in future research.

We were able to standardise the training and support in order to maximise treatment adherence and fidelity. However, the quality of support and training provided may have varied between sites, as it was delivered by a number of researchers with various levels of experience, skills, and qualifications. In terms of the extent to which the engagement strategies embedded into the trial (e.g., training, support, monitoring visits) were effective overall, data from the adherence questionnaires completed by caregivers at setup, MV1, and MV2 suggest the training and support provided by researchers was more than adequate; therefore, these components may not account for poor adherence.

Caregiver ratings of confidence in delivery and knowledge of iCST were very high and significantly improved from setup to MV2. In addition, we observed significant improvements in caregivers’ ability to apply the key principles and use the skills and techniques related to iCST. Despite this, caregivers did not perceive any corresponding improvements in the person with dementia’s engagement. From a fidelity perspective, these data suggest that caregivers felt they were delivering the intervention as intended and as they had been trained. However, a limitation of self-report methods such as questionnaires in lieu of more involved measures of fidelity such as researcher observation of sessions or audio/video recording is that it is difficult to closely monitor exactly what was delivered. In contrast with the findings from the training and monitoring visits, some caregivers who were interviewed post trial reported that they did not feel skilled enough to deliver the intervention and that the time between monitoring visits was too long. New paradigms in the design of pragmatic trials outline how “implementation errors” such as low treatment fidelity can be avoided [61].

Blinding to allocation appeared successful for the most part, with the majority of researchers reporting dyads equally likely to be in the iCST or TAU groups. Dyads were reminded not to disclose their allocation to visiting researchers. However, on rare occasions, they did share this information or left iCST materials on show, which were seen by the researcher during the 13-wk mid-point or post-test assessment visit. If researchers were unblinded at the 13-wk mid-point, they were typically advised not to conduct the post-test assessment if possible.

In terms of external validity, the intervention was tested in a wide diversity of urban and rural areas across eight sites in the UK. Despite this, there was a lack of variation in ethnicities of participants, which may affect scope for generalisation of results. Neither can we be sure the programme is suitable or acceptable across different cultures with the current data we have. The group CST programme has been successfully adapted for a wide range of cultures, with guidelines published to assist this process [62]; thus, iCST could be similarly tailored. Although, like CST, iCST was intended for people with mild to moderate dementia, most participants had mild dementia, and so it may be difficult to generalise the findings to people with moderate dementia. A further limitation of the trial is that the effect sizes for the significant improvements observed in relationship quality from the person with dementia’s perspective and caregiver QoL are small. Consequently, the extent to which the findings translate to tangible clinical or real-life benefits is hard to determine, particularly because investment of time and resources is necessary in order to deliver this intervention. However, there was a lot of feedback from people to indicate that the iCST activities were meaningful, enjoyable, and stimulating, suggesting that they may play a useful role as part of better care [60].

This trial contributes further to the body of knowledge of dyadic psychosocial interventions and demonstrates the benefits of relationship-centred care. The findings support the use of mentally stimulating activities as a means of improving outcomes for people with dementia and their caregivers [32,33]. Whilst CST is categorised as an intervention for people with dementia, the involvement of a family caregiver in the delivery of iCST and the observed positive impact of the sessions on both the person and the caregiver categorise iCST as a multi-component intervention. iCST provides caregivers with training and the manual with guidance and key principles that may be psychoeducational. iCST may also have elements of a support intervention, as caregivers were in regular telephone contact with researchers.

Improvements in the quality of the caregiving relationship were only demonstrated from the person with dementia’s perspective. Whilst caregiver QoL improved, the person with dementia’s QoL did not. This suggests that iCST may offer mutual, caregiver-specific, and patient-specific benefits. The sessions present opportunities for joint activities between the person and the caregiver, which may contribute to the positive outcomes each experienced.

Hellstrom, Nolan, and Lundh found caregivers and people with dementia identified four activities they felt “sustained couplehood”: “talking things through,” “being appreciative and affectionate,” “making the best of things,” and “keeping the peace” [32]. The iCST programme and key principles correspond to each of these needs, which may account for our findings. In terms of “talking things through” iCST activities facilitate discussion and may reinforce positive patterns of communication, which may transfer to interactions outside the sessions. Affection and appreciation may be demonstrated in the supportive and fun atmosphere that sessions are intended to create. Pairs may view participating in activities together as a source of enjoyment related to “making the best of things.” Finally, the iCST key principle of “focusing on opinions rather than facts” may contribute to “keeping the peace” by reducing criticism and celebrating success rather than dwelling on failure.

Providing enjoyable activities for a person with dementia can improve caregiver well-being [63]; thus, adopting a facilitative role in iCST sessions may be the mechanism responsible for the observed improvements in caregiver QoL. This appears plausible given our finding that caregivers who delivered more iCST sessions had fewer depressive symptoms.

The finding that the iCST programme did not significantly affect cognition contrasts with previous studies of group CST [13,15,26–28]. Furthermore, significant QoL benefits for people with dementia were not detected in this trial, despite being consistently associated with both short- and longer-term programmes of group CST [13,15,17]. The social setting and additional stimulation from participating in a group context may account for the difference in outcomes between iCST and group CST. Woods and colleagues suggest that the reported QOL benefits associated with CST are likely to be mediated by improvements in cognition [21]. Thus, the lack of significant cognitive change experienced by people with dementia may account for our lack of findings on QOL outcomes. Alternatively, an optimum “dose” of iCST may be necessary to impact cognition. There is evidence to suggest that two sessions of group CST per week are associated with cognitive benefits, whereas once weekly sessions are not [64]. Given 22% (39/180) of the sample did not complete any iCST sessions and adherence was generally low, people may not have received enough stimulation to benefit. The dyads who were either too busy or no longer wanted to participate amounted to just under 40% (18/46) of the total withdrawals, so in future studies, it is possible that with alternative approaches to retention and closer monitoring, some of these may have been persuaded to stay in the trial.

Given some people with dementia and their family caregivers did not fully engage in iCST, the trial could be replicated with enhanced processes to support better adherence. In the development phase of this trial, delivery by paid caregivers was suggested as an alternative if family caregivers were not willing or able to deliver iCST, and field-testing with a sample of paid caregivers demonstrated this was feasible [51]. Therefore, future research should investigate how far adherence can be improved if the intervention is delivered by a health care professional or paid caregiver.

Although iCST does not appear to deliver clinical benefits for cognition and QoL for people with dementia, there was evidence of improvement in terms of the caregiving relationship from the person with dementia’s perspective. There was also evidence of improvement in caregivers’ QoL and depressive symptoms for those who completed more sessions. From a clinical perspective, reduction in depressive symptoms and improved QoL of the caregiver by means of a low-cost, non-drug intervention are worthwhile outcomes. The longer-term associated effect of reducing depression may be that caregivers remain better mentally and perhaps physically (as a related consequence) for longer. This carries possible advantages such as prolonging their ability to provide care for the person with dementia, reduced instances of crises requiring intervention (e.g., emergency temporary hospitalisation), and less burden on health and social care services, as their health is maintained with less need for additional resources (e.g., medication, counselling, cognitive behavioural therapy). Enhancing the caregiving relationship through iCST may reduce the risk of presentation to services and deterioration of the person’s functioning, which has been associated with conflict in the caregiving relationship [10]. In turn, risk of institutionalisation may be reduced or delayed, which is important from a societal and cost perspective, as the cost of residential care is high [65]. This indicates that iCST may be introduced as a useful component of individually tailored home care packages, which may also help maintain people with dementia in their home situation for longer.

## Supporting information

S1 Appendix (DOC)

S1 CONSORT Checklist (DOC)

S1 iCST Materials list (DOC)

## Abbreviations

ADAS-Cog: Alzheimer’s Disease Assessment Scale–Cognitive Subscale
ANCOVA: analysis of covariance
BADLS: Bristol Activities of Daily Living Scale
BL: baseline
CD: compact disk
CDR: Clinical Dementia Rating Scale
CMHT: community mental health team
CST: cognitive stimulation therapy
DEMQoL: Dementia Quality of Life
EQ-5D: European Quality of Life–5 Dimensions
GDS-15: Geriatric Depression Scale
HADS: Hospital Anxiety and Depression Scale
iCST: individual cognitive stimulation therapy
ITT: intention to treat
MD: Mean difference
MMSE: Mini Mental State Examination
MV1: monitoring visit 1
MV2: monitoring visit 2
NHS: National Health Service
NPI: Neuropsychiatric Inventory
QCPR: Quality of the Carer–Patient Relationship
QoL: quality of life
QoL-AD: quality of life Alzheimer disease
RCT: randomised controlled trial
RS-14: Resilience Scale
SAE: serious adverse event
SD: standard deviation
SF-12: Short Form-12 Health Survey
TAU: treatment as usual
