# Supplementary material for: The impact of individual Cognitive Stimulation Therapy (iCST) on cognition, quality of life, caregiver health, and family relationships in dementia: A randomised controlled trial
Source: PLoS Med. 2017 Mar 28;14(3):e1002269. doi: 10.1371/journal.pmed.1002269 (PMC5369684; doi:10.1371/journal.pmed.1002269)
Supplement: S1 Appendix — (DOC) [file pmed.1002269.s001.doc]

| **Item** |  |
| --- | --- |
| **Name** | Individual Cognitive Stimulation Therapy for people with dementia (iCST) |
| **Why** | iCST is based on theoretical principles of reality orientation, and the same structure and principles of the group CST approach. Caregiver led iCST adapts a person-centered approach, in which the person is engaging in home-based cognitive stimulation activities. iCST activities focus on opportunities to express opinions and less on discussing factual information and place an emphasis on the person with dementia and their family caregiver spending enjoyable time together, using a specific framework of discussion. |
| **What** | ***Materials*** |
|  | 1. iCST Manual (guidance for caregivers) 2. iCST Activity Workbook (activities materials) 3. iCST Carer Diary (reporting and evaluating activities) 4. iCST Toolkit (boules, playing cards, dominoes, magnifying card, sound activity compact discs (CDs), colored pencils, and world and UK map) 5. iCST Training Pack (iCST role play exercises) 6. CST digital versatile disc (DVD) clips |
|  | ***Procedure***  Caregivers trained at their home, using a standardized training package aimed at demonstrating key principles of iCST. Additional support provided over the phone and additional home support visits. |
| **Who provided** | iCST was provided by family caregivers. Caregiver training and support was provided by the research team of unblinded researchers who were either mental health nurses, clinical psychologists, occupational therapists or research assistants. All unblinded researchers received standardized training in supporting family caregivers in iCST. |
| **How** | iCST was delivered by caregivers. iCST Caregiver Training was delivered by unblinded researchers. Unblinded researchers received training in a group. Support on iCST was provided as an additional home visit, and over the phone by unblinded researchers. |
| **Where** | iCST was delivered at the dyad’s home. |
| **When and How Much** | iCST consisted of 75 sessions delivered by the family caregiver for 30 minutes, for 3 times a week over 25 weeks. |
| **Tailoring** | Tailoring included additional home visits or telephone support, or provision of additional resources related to the intervention (i.e. books, DVD player) |
| **Modifications** | No modifications occurred during the intervention. |
| **How well** | Planned: Intervention compliance was assessed by self-reported questionnaires completed by caregivers and researchers.  Actual: Mean compliance was 31.6 (SD = 26.8) sessions, where 22% completed 0 sessions, 13% 1-10 sessions and 51% completed more than 30 sessions. |
| *SD=Standard deviation*  *Text A: TIDier checklist describing the iCST intervention* | |

| **iCST Session theme** | **Session number** |
| --- | --- |
| My life | 1, 2, 45, 46 |
| Current affairs | 3, 4, 57, 58 |
| Food | 5, 6, 55, 56 |
| Being creative | 7, 8, 63, 64 |
| Number games | 9, 10, 71, 72 |
| Quiz games | 11, 12, 75 |
| Sounds | 13, 14, 51, 52 |
| Physical games | 15. 16, 49, 50 |
| Categorising objects | 17, 18, 65, 66 |
| Household treasures | 19, 20 |
| Useful tips | 21, 22, 47, 48 |
| Thinking cards | 23, 24 |
| Visual clips discussion | 25, 26 |
| Art discussion | 27, 28, 43, 44 |
| Faces / Scenes | 29, 30, 59, 60 |
| Word games | 31, 32, 41, 42, 73, 74 |
| Slogans | 33, 34 |
| Association words discussion | 35, 36, 61, 62 |
| Orientation | 37, 38, 67, 68 |
| Using money | 39, 40, 69, 70 |
| Childhood | 53, 54 |

*iCST= Individual Cognitive Stimulation Therapy*

*Table A: Themes of iCST sessions and order of appearance in the manual*

***Intervention and control conditions***

Participants randomized to the intervention group received individual Cognitive Stimulation Therapy (iCST) at their own home. The control condition was ‘treatment as usual’ (TAU) with participants in this group receiving no additional intervention. The services and interventions available to people with dementia and family caregivers randomized to receive treatment as usual varied between and within the iCST centers and may have changed over time. We recorded use of drugs and services across the two groups and any changes that occurred. In general, services offered to the treatment as usual group were also available to those in the active treatment group condition.

It is very unlikely that any comparable (or even any other) individual cognitive stimulation intervention for the person with dementia would have been available, as these types of therapies are generally unavailable in the United Kingdom (UK). We followed standard best practice methods around pragmatic trials involving an intervention group as compared to usual care. Outside the iCST intervention both groups in general would have access to the same kind of mentally stimulating activities. It is possible that some participants in the treatment as usual group may have engaged in some form of mentally stimulating activities in day-centers, however this is unlikely to have been as structured as iCST. We asked sites to note instances where the person with dementia may have been engaged in cognitive stimulation groups by their local services. Those participants that have engaged in such activities during the three months prior to recruitment were considered ineligible.

*Text B: Excerpt from Health Technologies Assessment (HTA) report (Orgeta et al., 2015) describing treatment as usual (TAU) in the iCST trial.*

| **Characteristic**  ***Person with dementia*** | **Total (%)**  **(*n=*356)** | **Non-completers (%)**  **(*n=*83)** | **Completers (%)**  **(*n=*273)** |
| --- | --- | --- | --- |
| Female | 165 (46) | 39 (24) | 126 (76) |
| Ethnicity White | 331 (93) | 77 (23) | 254 (77) |
| Marital Status: married/cohabiting/civil partnership | 252 (71) | 59 (23) | 193 (77) |
| Lives with spouse/partner | 251 (71) | 59 (24) | 193 (77) |
| Highest level of education school leaver (14-16 years) | 213 (60) | 57 (27) | 156(73) |
| Anti-cholinesterase inhibitors | 270 (76) | 136 (76) | 159 (73) |
| Age | Mean (SD)  78.2 (7.49) | Mean (SD)  79.1 (8.52) | Mean (SD)  77.9 (7.14) |
|  |  |  |  |
| ***Caregiver***  Female | 261 (73) | 62 (24) | 199 (76) |
| Ethnicity White | 329 (92) | 75 (23) | 254 (77) |
| Marital Status: married/cohabiting/civil partnership | 297 (83) | 65 (22) | 232 (78) |
| Lives with spouse/partner | 289 (81) | 62(21) | 227 (79) |
| Highest level of education school leaver (14-16 years) | 159 (45) | 42(26) | 117 (74) |
| Age | Mean (SD)  65.7 (12.92) | Mean (SD)  65.5 (14.02) | Mean (SD)  65.8 (12.59) |
| *Table B:* Characteristics of people with dementia and caregivers who completed the trial and those who did not | | | |

| **Reason for drop outs* / withdrawals** | **Number of withdrawals iCST** | **Number of drop outs iCST** | **Total number of drop outs/withdrawals iCST** | **Number of withdrawals in TAU** | **Number of drop outs TAU** | **Total number of drop outs/withdrawals TAU** |
| --- | --- | --- | --- | --- | --- | --- |
| Dyad do not wish to continue | **13** | 0 | 13 | 4 | 0 | 4 |
| Carer unwell | 8 | 1 | 8 | 1 | 0 | 1 |
| Person with dementia unwell | 3 | 2 | 5 | 1 | 1 | 2 |
| Carer busy | 4 | 0 | 4 | 2 | 0 | 2 |
| Person with dementia deteriorated | 3 | 0 | 3 | 1 | 0 | 1 |
| Residential care | 3 | 0 | 3 | 3 | 0 | 3 |
| Dyad are abroad / on holiday | 1 | 1 | 2 | 0 | 0 | 0 |
| Family crisis | 0 | 2 | 2 | 1 | 0 | 1 |
| Unable to contact dyad | 2 | 0 | 2 | 2 | 3 | 5 |
| Cross recruitment | 1 | 0 | 1 | 0 | 0 | 0 |
| Moved out of area | 1 | 0 | 1 | 0 | 0 | 0 |
| Carer no longer caring for person with dementia | 1 | 0 | 1 | 0 | 0 | 0 |
| Person with dementia depressed | 1 | 0 | 1 | 0 | 1 | 1 |
| Carer diagnosed with dementia | 1 | 0 | 1 | 0 | 0 | 0 |
| Carer stress | 1 | 0 | 1 | 0 | 0 | 0 |
| Carer burden | 1 | 0 | 1 | 0 | 0 | 0 |
| Person with dementia moved (still in local area) | 0 | 1 | 1 | 0 | 0 | 0 |
| Person with dementia hospitalized | 0 | 1 | 1 | 0 | 1 | 1 |
| Person with dementia died | 1 | 0 | 1 | **6** | 0 | 6 |
| Person with dementia does not wish to continue | 1 | 0 | 1 | 1 | 0 | 1 |
| Carer died | 0 | 0 | 0 | 1 | 0 | 1 |
| Dyad unhappy with allocation | 0 | 0 | 0 | **6** | 0 | 6 |
| Person with dementia has diagnosis of a significant health condition | 0 | 0 | 0 | 2 | 0 | 2 |
| Carer depressed | 0 | 0 | 0 | 1 | 0 | 1 |
| Dyad both unwell | 0 | 0 | 0 | 1 | 0 | 1 |
| Dyad relationship breakdown | 0 | 0 | 0 | 1 | 0 | 1 |
| Person with dementia stressed | 0 | 0 | 0 | 1 | 0 | 1 |
| Person with dementia lacks mental capacity | 0 | 0 | 0 | 1 | 0 | 1 |
| Person refused assessment | 0 | 0 | 0 | 0 | 1 | 1 |
| Carer thinks participating in iCST trial has a negative impact on person with dementia | 0 | 0 | 0 | 0 | 1 | 1 |
| **Total** | **46** | **8** | **54** | **36** | **8** | **44** |

*iCST= individual Cognitive Stimulation Therapy, TAU= Treatment as usual*

*Table C: Reasons for drop outs and withdrawals in the iCST and TAU groups * drop outs defined as failure to complete an assessment at mid-point. Withdrawals defined as ceasing to continue participating in the trial.*

|  | **Actual Treatment allocation** | | |
| --- | --- | --- | --- |
| **Researcher rating** | **iCST (%)** | **TAU (%)** | **Total (%)** |
| Correct ‘Definite’ judgment | 22 (19) | 4 (3) | 26 (10) |
| Correct ‘More likely’ judgment: | 17 (15) | 17 (12) | 34 (13) |
| Equally likely to be in iCST or TAU | 65 (57) | 80 (57) | 145 (57) |
| Incorrect ‘More likely’ judgment: | 10 (9) | 31 (22) | 41 (16) |
| Incorrect ‘Definite’ judgment: | 0 | 9 (6) | 9 (4) |
| Total | *114* | *141* | *255* |

*iCST=individual Cognitive Stimulation Therapy, TAU=Treatment as usual*

|  | **Actual Treatment allocation** | | |
| --- | --- | --- | --- |
| **Researcher rating** | **iCST (%)** | **TAU (%)** | **Total (%)** |
| Correct ‘Definite’ judgment | 13 (12) | 6 (4) | 19 (7) |
| Correct ‘More likely’ judgment: | 14 (13) | 28 (18) | 42 (16) |
| Equally likely to be in iCST or TAU | 68 (65) | 92 (58) | 160 (60) |
| Incorrect ‘More likely’ judgment: | 11 (10) | 20 (13) | 31 (12) |
| Incorrect ‘Definite’ judgment: | 0 | 12 (7) | 12 (5) |
| Total | *106* | *158* | *264* |

*Table D: Researchers’ perceived group allocation at 13 week mid-point assessment (n=*264*)*

*iCST= individual Cognitive Stimulation Therapy, TAU=Treatment as usual*

*Table E: Researchers’ perceived group allocation at post-test assessment (n=255)*

|  | ***Baseline*** | | | | ***13 weeks (mid-point)*** | | | | ***26 weeks (post-test)*** | | | |
| --- | --- | --- | --- | --- | --- | --- | --- | --- | --- | --- | --- | --- |
| ***Outcome measure*** | ***N***  ***Mis.*** | ***iCST***  ***N=180***  ***Mean (SD)*** | ***N***  ***Mis.*** | ***TAU***  ***N=176***  ***Mean (SD)*** | ***N***  ***Mis.*** | ***iCST***  ***N=142***  ***Mean (SD)*** | ***N***  ***Mis.*** | ***TAU***  ***N=146***  ***Mean (SD)*** | ***N***  ***Mis.*** | ***iCST***  ***N=134***  ***Mean (SD)*** | ***N***  ***Mis.*** | ***TAU***  ***N=139***  ***Mean (SD)*** |
| ***Person with dementia*** |  |  |  |  |  |  |  |  |  |  |  |  |
| ADAS Cog | 1 | 21.47(9.22) | 1 | 19.79 (8.03) | 4 | 20.86 (9.73) | 6 | 19.50 (8.97) | 6 | 20.69 (9.39) | 5 | 20.39 (9.91) |
| QoL-AD |  | 38.01 (5.44) |  | 37.96 (6.04) | 2 | 37.90 (5.52) | 2 | 38.09 (5.63) | 5 | 37.86 (5.13) | 1 | 37.71 (5.91) |
| DemQoL | 3 | 93.85 (11.76) | 3 | 92.18 (13.55) | 7 | 94.08 (10.92) | 4 | 94.05 (11.80) | 6 | 95.46 (11.17) | 3 | 95.12 (11.11) |
| NPI total |  | 11.21 (13.96) |  | 10.99 (11.98) | 2 | 10.67 (13.30) |  | 12.07 (12.61) | 1 | 11.57 (13.72) | 1 | 11.59 (12.80) |
| GDS 15 | 3 | 3.14 (2.64) | 3 | 3.16 (3.15) | 9 | 2.98 (2.56) | 3 | 3.03 (2.86) | 8 | 2.90 (2.55) | 3 | 2.85 (2.67) |
| QCPR total | 6 | 55.17 (8.89) | 2 | 56.72 (8.73) | 4 | 56.30 (8.98) | 3 | 55.82 (9.06) | 3 | 56.88 (8.59) | 1 | 55.55 (10.25) |
| QCPR Warmth | 1 | 33.19 (5.08) | 1 | 33.98 (5.20) | 1 | 33.32 (5.49) |  | 33.25 (5.38) | 1 | 33.78 (4.97) |  | 33.07 (5.92) |
| QCPR Criticism & conflict | 1 | 22.07 (4.78) | 1 | 22.69 (4.66) | 1 | 22.80 (4.46) |  | 22.54 (4.75) |  | 22.95 (4.59) |  | 22.46 (5.15) |
| MMSE |  | 21.12 (4.48) |  | 21.33 (4.11) | 1 | 20.59 (5.02) | 2 | 20.89 (4.83) | 4 | 20.68 (4.76) | 1 | 21.19 (5.21) |
| BADLS [P] |  | 5.16 (5.45) |  | 4.49 (4.09) |  | 14.53 (10.34) | 1 | 13.55 (8.20) | 3 | 15.39 (10.78) | 1 | 14.56 (8.86) |
| QoL-AD [P] | 0 | 32.88 (6.83) |  | 33.09 (6.22) | 1 | 32.64 (6.25) | 2 | 31.93 (5.84) | 1 | 32.46 (6.20) |  | 31.99 (6.30) |
| DemQoL [P] | 1 | 97.99 (13.17) | 2 | 98.59 (12.76) | 2 | 99.26 (12.38) | 1 | 98.75 (11.96) | 1 | 99.42 (12.41) | 1 | 98.18 (12.80) |
| **Caregiver** |  |  |  |  |  |  |  |  |  |  |  |  |
| SF12 Physical component | 1 | 51.46 (10.25) | 1 | 50.20 (10.32) |  | 51.13 (9.73) |  | 49.97 (10.34) |  | 50.06 (10.53) | 1 | 48.63 (10.87) |
| SF12 Mental component | 1 | 49.42 (8.10) | 1 | 48.14 (9.42) |  | 47.98 (9.90) | 2 | 47.93 (9.96) |  | 48.88 (9.41) |  | 47.88 (10.13) |
| HADS total | 3 | 9.63 (6.11) |  | 10.02 (6.67) | 1 | 10.37 (6.98) |  | 10.41 (6.89) | 1 | 10.06 (6.92) |  | 11.16 (7.59) |
| HADS Anxiety | 3 | 5.84 (3.58) |  | 6.03 (3.88) | 1 | 6.33 (4.35) |  | 6.06 (4.00) | 1 | 6.03 (4.32) |  | 6.36 (4.44) |
| HADS Depression | 3 | 3.79 (3.30) |  | 3.99 (3.40) | 1 | 4.03 (3.30) |  | 4.36 (3.48) | 1 | 4.03 (3.29) |  | 4.80 (3.81) |
| EQ5D health state today | 3 | 78.37 (16.63) |  | 76.24 (19.28) | 1 | 78.13 (16.84) |  | 76.45 (16.93) |  | 78.63 (17.09) |  | 76.58 (16.90) |
| EQ5D calculated utility value | 1 | 0.83 (0.20) |  | 0.81 (0.21) | 1 | 0.82 (0.20) |  | 0.79 (0.22) | 2 | 0.82 (0.21) |  | 0.75 (0.25) |
| RS 14 | 2 | 83.39 (10.98) | 1 | 83.63 (10.43) |  | 83.27 (11.14) |  | 83.49 (10.47) | 1 | 83.44 (10.86) |  | 81.83 (12.90) |
| NPI Carer distress |  | 3.15 (2.67) |  | 3.23 (2.59) | 2 | 3.13 (2.48) |  | 3.18 (2.39) | 1 | 3.11 (2.65) | 1 | 3.25 (2.41) |
| QCPR total | 3 | 59.21 (6.67) | 3 | 58.21 (6.63) | 8 | 60.12 (6.33) | 4 | 59.73 (6.67) | 9 | 60.14 (6.54) | 4 | 59.76 (6.77) |
| QCPR Warmth | 3 | 34.94 (3.77) | 3 | 34.59 (3.64) | 8 | 35.20 (3.66) | 4 | 35.69 (3.45) | 8 | 35.25 (3.57) | 4 | 34.95 (3.51) |
| QCPR Criticism & conflict | 3 | 24.26 (3.63) | 2 | 23.63 (3.81) | 8 | 24.92 (3.57) | 4 | 24.04 (4.41) | 9 | 24.90 (3.66) | 4 | 24.81 (4.01) |
| *iCST= individual Cognitive Stimulation Therapy group, SD= Standard deviation, [P]=Proxy rated measure, Mis.= missing data, TAU=Treatment as usual group, ADAS-Cog= Alzheimer’s Disease Assessment Scale-Cognitive, QoL-AD= Quality of Life Alzheimer’s Disease, DEMQoL=Dementia Quality of Life, NPI= Neuropsychiatric Inventory, GDS= Geriatric Depression Scale, QCPR= Quality of the Caregiving Relationship, MMSE= Mini-Mental State Examination, BADLS= Bristol Activities of Daily Living Scale, SF12=Short Form Survey, HADS=Hospital Anxiety & Depression Scale, EQ-5D=EuroQoL, RS 14= Resilience Scale*  *Table F: Unadjusted means for each of the outcome measures for iCST and TAU at 13 week mid-point & 26 week post test.* | | | | | | | | | | | | |

|  | Baseline | | 13 week mid-point | | 26 week post test | |
| --- | --- | --- | --- | --- | --- | --- |
|  | ***iCST***  ***N=180***  ***Mean*** | ***TAU***  ***N=176***  ***Mean*** | ***iCST***  ***N=142***  ***Mean change from baseline (SD)*** | ***TAU***  ***N=146***  ***Mean change from baseline (SD)*** | ***iCST***  ***N=134***  ***Mean change from baseline (SD)*** | ***TAU***  ***N=139***  ***Mean change from baseline (SD)*** |
| ***Person with dementia*** |  |  |  |  |  |  |
| ADAS Cog | 21.47 | 19.79 | -0.29 (5.87) | 0.03 (6.16) | 0.33 (5.65) | 1.24 (5.94) |
| QoL-AD | 38.01 | 37.96 | -0.36 (5.01) | -0.13 (4.6) | -0.70 (4.07) | -0.43 (5.13) |
| DemQoL | 93.85 | 92.18 | -0.47 (7.59) | 0.32 (10.85) | 1.09 (7.2) | 1.56 (10.39) |
| NPI total | 11.21 | 10.99 | 0.21 (11.05) | 1.96 (10.08) | 1.14 (11.72) | 1.40 (10.1) |
| GDS 15 | 3.14 | 3.16 | -0.01 (1.98) | 0.11 (2.24) | -0.11 (1.94) | -0.09 (2.7) |
| QCPR total | 55.17 | 56.72 | 0.28 (5.62) | -0.94 (5.39) | 0.40 (5.99) | -1.46 (6.9) |
| QCPR Warmth | 33.19 | 33.98 | -0.26 (3.88) | -0.75 (3.49) | -0.15 (3.6) | -1.02 (4.24) |
| QCPR Criticism & conflict | 22.07 | 22.69 | 0.54 (3.51) | -0.18 (3.13) | 0.53 (4.05) | -0.43 (3.7) |
| MMSE | 21.12 | 21.33 | -0.46 (3.18) | -0.87 (3.5) | -0.96 (2.99) | -0.67 (3.62) |
| BADLS [P] | 5.16 | 4.49 | 9.51 (6.23) | 9.35 (5.91) | 10.52 (6.97) | 10.55 (6.54) |
| QoL-AD [P] | 32.88 | 33.09 | -0.62 (5.49) | -1.40 (4.54) | -1.06 (5.99) | -1.49 (4.95) |
| DemQoL [P] | 97.99 | 98.59 | 0.84 (10.77) | 0.17 (11.46) | 1.70 (12.06) | -0.41 (9.96) |
| **Caregiver** |  |  |  |  |  |  |
| SF12 Physical component | 51.46 | 50.20 | -0.78 (5.98) | -0.45 (6.72) | -1.85 (7.18) | -2.13 (7.32) |
| SF12 Mental component | 49.42 | 48.14 | -1.56 (6.91) | -0.54 (7.42) | -0.92 (7.05) | -0.57 (8.43) |
| HADS total | 9.63 | 10.02 | 0.86 (4.36) | 0.62 (4.3) | 0.75 (4.52) | 1.37 (5.27) |
| HADS Anxiety | 5.84 | 6.03 | 0.51 (2.75) | 0.19 (2.65) | 0.26 (2.95) | 0.43 (3.25) |
| HADS Depression | 3.79 | 3.99 | 0.35 (2.36) | 0.44 (2.36) | 0.49 (2.33) | 0.94 (2.72) |
| EQ5D health state today | 78.37 | 76.24 | -1.36 (13.3) | -0.83 (17.23) | -0.68 (13.64) | -0.91 (18.22) |
| EQ5D calculated utility value | 0.83 | 0.81 | -0.01 (0.19) | -0.04 (0.15) | -0.02 (0.2) | -0.07 (0.2) |
| RS 14 | 83.39 | 83.63 | -0.15 (7.47) | -0.14 (6.45) | -0.20 (7.2) | -1.78 (9.17) |
| NPI Carer distress | 3.15 | 3.23 | 0.13 (2.21) | 0.09 (2.19) | 0.11 (2.25) | 0.17 (2.08) |
| QCPR total | 59.21 | 58.21 | 0.23 (6.71) | 0.77 (7.11) | -0.15 (5.78) | 1.12 (6.64) |
| QCPR Warmth | 34.94 | 34.59 | -0.12 (4.04) | 0.87 (3.84) | -0.30 (3.4) | 0.21 (3.8) |
| QCPR Criticism & conflict | 21.47 | 23.63 | 0.36 (3.81) | -0.09 (4.43) | 0.17 (3.17) | 0.89 (3.88) |
| *iCST= individual Cognitive Stimulation Therapy group, SD= Standard deviation, [P]=Proxy rated measure, TAU=Treatment as usual group, ADAS-Cog= Alzheimer’s Disease Assessment Scale-Cognitive, QoL-AD= Quality of Life Alzheimer’s Disease, DEMQoL=Dementia Quality of Life, NPI= Neuropsychiatric Inventory, GDS= Geriatric Depression Scale, QCPR= Quality of the Caregiving Relationship, MMSE= Mini-Mental State Examination, BADLS= Bristol Activities of Daily Living Scale, SF12=Short Form Survey, HADS=Hospital Anxiety & Depression Scale, EQ-5D=EuroQoL, RS 14= Resilience Scale*  *Table G: Change from baseline for each of the outcome measures for iCST and TAU at 13 week mid-point & 26 week post test.* | | | | | | |

| **Measure** | **Observed data** | |  |  | **Imputed data** | |  |  |  |  |  |  |
| --- | --- | --- | --- | --- | --- | --- | --- | --- | --- | --- | --- | --- |
| **13 week mid-point** |  |  |  |  |  |  | **Median F** |  | **Low F** |  | **High F** |  |
| **Person with dementia** | **Coefficient** | **SE** | ***F*** | ***p* value** | **Pooled coefficient** | **SE** | ***F*** | ***p* value** | ***F*** | ***p* value** | ***F*** | ***p* value** |
| ADAS-Cog | 0.006 | 0.030 | 0.042 | 0.838 | 0.002 | 0.030 | 0.038 | 0.846 | 0.005 | 0.946 | 0.071 | 0.790 |
| QoL-AD | 0.019 | 0.021 | 0.866 | 0.353 | 0.019 | 0.021 | 0.824 | 0.365 | 0.685 | 0.409 | 1.015 | 0.315 |
| DEMQoL | -0.021 | 0.042 | 0.246 | 0.620 | -0.020 | 0.042 | 0.190 | 0.663 | 0.122 | 0.727 | 0.408 | 0.524 |
| NPI total | -0.045 | 0.048 | 0.880 | 0.349 | -0.046 | 0.048 | 0.903 | 0.343 | 0.877 | 0.350 | 0.916 | 0.339 |
| GDS-15 | -0.003 | 0.010 | 0.076 | 0.783 | -0.003 | 0.010 | 0.055 | 0.815 | 0.000 | 0.986 | 0.628 | 0.429 |
| QCPR total* | 0.049 | 0.026 | 3.458 | 0.064 | 0.049 | 0.026 | 3.495 | 0.063 | 3.468 | 0.064 | 3.546 | 0.061 |
| QCPR warmth | 0.003 | 0.018 | 0.036 | 0.850 | 0.003 | 0.018 | 0.033 | 0.856 | 0.031 | 0.859 | 0.043 | 0.836 |
| QCPR criticism & conflict*^,+^ | 0.043 | 0.015 | 8.268 | 0.004 | 0.043 | 0.015 | 8.383 | 0.004 | 8.377 | 0.004 | 8.386 | 0.004 |
| MMSE | 0.026 | 0.016 | 2.667 | 0.104 | 0.026 | 0.016 | 2.764 | 0.098 | 2.419 | 0.121 | 2.861 | 0.092 |
| BADLS (P)^+^ | 0.024 | 0.029 | 0.671 | 0.413 |  |  |  |  |  |  |  |  |
| QoL-AD (P) | 0.038 | 0.022 | 3.015 | 0.084 | 0.037 | 0.022 | 2.833 | 0.093 | 2.782 | 0.096 | 3.181 | 0.076 |
| DEMQoL (P) | 0.019 | 0.048 | 0.155 | 0.694 | 0.019 | 0.048 | 0.160 | 0.689 | 0.137 | 0.711 | 0.195 | 0.659 |
| **Caregiver** |  |  |  |  |  |  |  |  |  |  |  |  |
| SF-12 PCS | 0.015 | 0.030 | 0.270 | 0.604 |  |  |  |  |  |  |  |  |
| SF-12 MCS | 0.007 | 0.034 | 0.040 | 0.843 |  |  |  |  |  |  |  |  |
| HADS total | -0.024 | 0.021 | 1.271 | 0.260 | -0.023 | 0.021 | 1.235 | 0.267 | 1.219 | 0.271 | 1.310 | 0.253 |
| HADS (anxiety) | -0.012 | 0.013 | 0.806 | 0.370 | -0.011 | 0.013 | 0.752 | 0.387 | 0.716 | 0.398 | 0.847 | 0.358 |
| HADS (depression) | -0.014 | 0.011 | 1.519 | 0.219 | -0.014 | 0.011 | 1.496 | 0.222 | 1.450 | 0.230 | 1.667 | 0.198 |
| EQ-5D health state 0.891today | 0.126 | 0.066 | 3.689 | 0.056 | 0.127 | 0.066 | 3.773 | 0.053 | 3.582 | 0.059 | 3.796 | 0.052 |
| EQ-5D calculated utility value | 0.001 | 0.001 | 2.573 | 0.110 | 0.001 | 0.001 | 2.566 | 0.110 | 2.558 | 0.111 | 2.607 | 0.108 |
| RS-14 | -0.031 | 0.033 | 0.891 | 0.346 |  |  |  |  |  |  |  |  |
| NPI (carer distress) | -0.007 | 0.009 | 0.658 | 0.418 | -0.007 | 0.009 | 0.646 | 0.422 | 0.598 | 0.440 | 0.746 | 0.389 |
| QCPR total | -0.004 | 0.030 | 0.022 | 0.883 | -0.003 | 0.030 | 0.033 | 0.855 | 0.002 | 0.968 | 0.088 | 0.766 |
| QCPR (warmth) | -0.008 | 0.017 | 0.223 | 0.637 | -0.006 | 0.017 | 0.074 | 0.786 | 0.020 | 0.888 | 0.466 | 0.495 |
| QCPR (criticism & conflict) | 0.005 | 0.019 | 0.061 | 0.806 | 0.004 | 0.019 | 0.070 | 0.792 | 0.001 | 0.980 | 0.206 | 0.650 |
| *iCST= individual Cognitive Stimulation Therapy group, SE= Standard error, [P]=Proxy rated measure, F = F statistic, TAU=Treatment as usual group, ADAS-Cog= Alzheimer’s Disease Assessment Scale-Cognitive, QoL-AD= Quality of Life Alzheimer’s Disease, DEMQoL=Dementia Quality of Life, NPI= Neuropsychiatric Inventory, GDS= Geriatric Depression Scale, QCPR= Quality of the Caregiving Relationship, MMSE= Mini-Mental State Examination, BADLS= Bristol Activities of Daily Living Scale, SF12=Short Form Survey, HADS=Hospital Anxiety & Depression Scale, EQ-5D=EuroQoL, RS 14= Resilience Scale*  *Table H: Regression coefficient (and Standard Error [SE]) of the association between each person with dementia and caregiver outcome measure and the number of sessions of iCST attended at 13 week mid-point after adjusting for the baseline outcome measures*  ** Significant difference*  *+ No missing data so imputed data rows left blank* | | | | | | | | | | | | |
