# Supplementary material for: The impact of individual Cognitive Stimulation Therapy (iCST) on cognition, quality of life, caregiver health, and family relationships in dementia: A randomised controlled trial
Source: PLoS Med. 2017 Mar 28;14(3):e1002269. doi: 10.1371/journal.pmed.1002269 (PMC5369684; doi:10.1371/journal.pmed.1002269)
Supplement: S1 iCST Materials list — (DOC) [file pmed.1002269.s003.doc]

***iCST materials list***

iCST Manual (see supplementary file)

iCST Activity Workbook (see supplementary file)

UK map

World map

Playing cards

Dominoes

Colored pencils

Magnifying card

Sounds CD (session 13)

Sounds CD (session 14)

Sounds CD (session 51)

Set of boules
